# Supplementary material for: Psychosocial Processes in Healthcare Workers: How Individuals’ Perceptions of Interpersonal Communication Is Related to Patient Safety Threats and Higher-Quality Care
Source: Int J Environ Res Public Health. 2023 May 1;20(9):5698. doi: 10.3390/ijerph20095698 (PMC10178743; doi:10.3390/ijerph20095698)
Supplement: Supplementary file 1 [file ijerph-20-05698-s001.zip › ijerph-2344999-supplementary.pdf]

Psychosocial Processes in Healthcare Workers: How Individuals' Perceptions of Interpersonal Communication Is Related to Patient Safety Threats and Higher-Quality Care

Supplementary Tables with translated measures (used items were worded in German)

Table S1: Measurement of Psychological safety

Scale Introduction	Item
Please think more in detail about your team: How much do the following statements apply to you?	Working with members of my team, my unique skills and talents are valued and utilized.
	Members of my team are able to bring up problems and tough issues.
	No one on my team would deliberately act in a way that undermines my efforts.
	It is difficult to ask other members of my team for help.

Note. All items were measured with a six-point Likert scale with the answer options '1' (*not at all*) ranging to '6' (*absolutely*). Adapted scale by Edmondson, A. C. (1999). Psychological safety and learning behavior in work teams. *Administrative Science Quarterly, 44*(2), 350–383.

Psychosocial Processes in Healthcare Workers: How Individuals' Perceptions of Interpersonal Communication Is Related to Patient Safety Threats and Higher-Quality Care

Table S2: Measurement of interpersonal communication competencies (study 1: online survey)

Scale Introduction	Item
	I answer all patient's questions sufficiently.
	When I explain the treatment or procedure, I am sure that my explanations are completely correct.
	I explain all examinations or procedures in such way that my patients understand them.
	I also involve the persons accompanying the patients or the patients themselves, into the decision-making process.
	I take concerns and fears seriously.
	I consider how much prior knowledge a patient has and how much he can understand.
	In my day-to-day work I can deal well with language barriers on the part of patients and accompanying persons.
	In general, I am satisfied with my level of communication.

Note. All items were measured with a six-point Likert scale with the answer options '1' (*not at all*) ranging to '6' (*absolutely*). Newly devised scale based on Rider, E. A., & Keefer, C. H. (2006). Communication skills competencies: Definitions and a teaching toolbox. *Medical Education, 40*(7), 624–629. https://doi.org/10.1111/j.1365-2929.2006.02500.x

Psychosocial Processes in Healthcare Workers: How Individuals' Perceptions of Interpersonal Communication Is Related to Patient Safety Threats and Higher-Quality Care

Table S3: Measurement of interpersonal communication competencies (study 2: hospital survey)

Scale Introduction	Item
**We as a team …**<br><br>*"We" means you and your colleagues, i.e., those with whom you normally work in a team (not just your own professional group).*	…answer all patients' questions sufficiently.
	… explain treatments or procedures, in such a way that we are sure that the explanation is completely correct.
	… explain all examinations or procedures in such a way that the patients understand them.
	… also involve the people accompanying the patients in decisions.
	… listen to the patients' concerns and fears.
	… consider how much prior knowledge a patient has and how much they can understand.
	… can deal well with language barriers on the part of patients and accompanying persons in our everyday work.

Note. All items were measured with a six-point Likert scale with the answer options '1' (*not at all*) ranging to '6' (*absolutely*). Newly devised scale based on Rider, E. A., & Keefer, C. H. (2006). Communication skills competencies: Definitions and a teaching toolbox. *Medical Education, 40*(7), 624–629. https://doi.org/10.1111/j.1365-2929.2006.02500.x

Psychosocial Processes in Healthcare Workers: How Individuals' Perceptions of Interpersonal Communication Is Related to Patient Safety Threats and Higher-Quality Care

Table S4: Measurement of patient safety threats

Scale Introduction	Item
On a usual workday, I usually notice that …	… colleagues have poorly washed or disinfected their hands.
	… there were conflicts among colleagues.
	… patients were poorly informed about examinations or treatment procedures.
	… general applicable due diligence obligations were violated.
	… colleagues have forgotten to disclose important information.
	… my opinions and suggestions were ignored.
	… important information (e.g., about allergies) was missing from a patient's record.
	… processes did not work smoothy.
	… colleagues or I were emotionally burdened.
	… colleagues or I had insufficient knowledge of technical equipment.
	… colleagues or I were inadequately prepared for discussions with patients.
	… not sufficiently important or useful treatment options (e.g., medication, physiotherapy, etc.) were considered.
	… important information was missing from patients' discharge reports.
	… the cooperation between the hospital and external treatment providers did not work properly.
	… conflicts have arisen due to language barriers.

Note. All items were measured with a six-point Likert scale with the answer options '1' (*not at all*) ranging to '6' (*absolutely*). Adapted scale by Keller, F. M., Derksen, C., Kötting, L., Schmiedhofer, M., & Lippke, S. (2021). Development of the perceptions of preventable adverse events assessment tool (PPAEAT): measurement properties and patients' mental health status. *International Journal for Quality in Health Care*, *33*(2)

Psychosocial Processes in Healthcare Workers: How Individuals' Perceptions of Interpersonal Communication Is Related to Patient Safety Threats and Higher-Quality Care

Table S5: Measurement of quality of care (study 1: online survey)

Scale Introduction	Item
Please rate below how you perceive your daily work routine based on the given answer options.	Communication and coordination between the various professional groups work well.
	It is always possible for me to get current information about patients' health status, treatments, or test results.
	I am informed about admission, transfer, and discharge of patients in a timely manner.
	Patients and their accompanying persons are treated with attention and consideration.
	The areas where I work, most often with female patients, are occasionally dirty or in need of cleaning.
	I have the opportunity to take advantage of interesting and current professional development opportunities.
	My working schedule takes individual wishes into account.
	Trainees or new employees have a designated contact person.
	I consider that mistakes and complaints are dealt with in a responsible manner in our hospital.
	My working schedule is burdening.
	Confusion often arises as to who is responsible for which patient.
	In my field of work, tasks are clearly defined.

Note. All items were measured with a six-point Likert scale with the answer options '1' (*not at all*) ranging to '6' (*absolutely*). Newly devised scale based on Ibn El Haj, H.; Lamrini, M.; Rais, N. Quality of care between Donabedian model and ISO9001V2008. *Int. J. Qual. Res.* 2013, *7*(1), 17–30. Klein, J., Grosse Frie, K., Blum, K., & von dem Knesebeck, O. (2011). Psychosocial stress at work and perceived quality of care among clinicians in surgery. *BMC Health Services Research*, *11*, 109. https://doi.org/10.1186/1472-6963-11-109. Winkler, H., & Prinz, A. (2009). Zertifizierung von Einrichtungen der Sucht-Rehabilitation–Erwartungen und Erfolge [Certification of addiction rehabilitation facilities–expectations and successes]. *SUCHT*, *55*(3), 164–168. https://doi.org/10.1024/2009.03.05

Psychosocial Processes in Healthcare Workers: How Individuals' Perceptions of Interpersonal Communication Is Related to Patient Safety Threats and Higher-Quality Care

Table S6: Measurement of quality of care (study 2: hospital survey; short scale)

Scale Introduction	Item
Please rate below how you perceive your daily work routine based on the given answer options.	Communication and coordination between the various professional groups work well.
	It is always possible for me to get current information about patients' health status, treatments, or test results.
	I am informed about admission, transfer, and discharge of patients in a timely manner.
	Patients and their accompanying persons are treated with attention and consideration.
	The areas where I work, most often with female patients, are occasionally dirty or in need of cleaning.
	I have the opportunity to take advantage of interesting and current professional development opportunities.
	My working schedule takes individual wishes into account.
	Trainees or new employees have a designated contact person.

Note. All items were measured with a six-point Likert scale with the answer options '1' (*not at all*) ranging to '6' (*absolutely*). Newly devised scale based on Hall, W., Violato, C., Lewkonia, R., Lockyer, J., Fidler, H., Toews, J., & Moores, D. (1999). *Assessment of physician performance in Alberta: The physician achievement review. Cmaj, 1*(161), 52–57. Klein, J., Grosse Frie, K., Blum, K., & von dem Knesebeck, O. (2011). Psychosocial stress at work and perceived quality of care among clinicians in surgery. *BMC Health Services Research, 11*, 109. https://doi.org/10.1186/1472-6963-11-109. Winkler, H., & Prinz, A. (2009). Zertifizierung von Einrichtungen der Sucht-Rehabilitation–Erwartungen und Erfolge [Certification of addiction rehabilitation facilities–expectations and successes]. *SUCHT, 55*(3), 164–168. https://doi.org/10.1024/2009.03.05
